# Population-Based Biomonitoring of Exposure to Organophosphate and Pyrethroid Pesticides in New York City

**DOI:** 10.1289/ehp.1206015

**Published:** 2013-09-27

**Authors:** Wendy McKelvey, J. Bryan Jacobson, Daniel Kass, Dana Boyd Barr, Mark Davis, Antonia M. Calafat, Kenneth M. Aldous

**Affiliations:** 1 Division of Environmental Health, New York City Department of Health and Mental Hygiene, New York, New York, USA; 2 Department of Environmental Health, Rollins School of Public Health, Emory University, Atlanta, Georgia, USA; 3 National Center for Environmental Health, Centers for Disease Control and Prevention, Atlanta, Georgia, USA; 4 Laboratory of Organic Analytical Chemistry, Division of Environmental Health Sciences, Wadsworth Center, New York State Department of Health, Albany, New York, USA

## Abstract

Background: Organophosphates and pyrethroids are the most common classes of insecticides used in the United States. Widespread use of these compounds to control building infestations in New York City (NYC) may have caused higher exposure than in less-urban settings.

Objectives: The objectives of our study were to estimate pesticide exposure reference values for NYC and identify demographic and behavioral characteristics that predict exposures.

Methods: The NYC Health and Nutrition Examination Survey was a population-based, cross-sectional study conducted in 2004 among adults ≥ 20 years of age. It measured urinary concentrations of organophosphate metabolites [dimethylphosphate (DMP), dimethylthiophosphate (DMTP), dimethyldithiophosphate, diethylphosphate, diethylthiophosphate, and diethyldithiophosphate] in 883 participants, and pyrethroid metabolites [3-phenoxybenzoic acid (3-PBA), *trans*-3-(2,2-dichlorovinyl)-2,2-dimethylcyclopropane-1-carboxylic acid (*trans*-DCCA), 4-fluoro-3-phenoxybenzoic acid, and *cis*-3-(2,2-dibromovinyl)-2,2-dimethylcyclopropane-1-carboxylic acid] in 1,452 participants. We used multivariable linear regression to estimate least-squares geometric mean total dialkylphospate (ΣDAP) and 3-PBA concentrations across categories of predictors.

Results: The dimethyl organophosphate metabolites had the highest 95th percentile concentrations (87.4 μg/L and 74.7 μg/L for DMP and DMTP, respectively). The highest 95th percentiles among pyrethroid metabolites were measured for 3-PBA and *trans*-DCCA (5.23 μg/L and 5.94 μg/L, respectively). Concentrations of ΣDAP increased with increasing age, non-Hispanic white or black compared with Hispanic race/ethnicity, professional pesticide use, and increasing frequency of fruit consumption; they decreased with non-green vegetable consumption. Absolute differences in geometric mean urinary 3-PBA concentrations across categories of predictors were too small to be meaningful.

Conclusion: Estimates of exposure to pyrethroids and dimethyl organophosphates were higher in NYC than in the United States overall, underscoring the importance of considering pest and pesticide burdens in cities when formulating pesticide use regulations.

Citation: McKelvey W, Jacobson JB, Kass D, Barr DB, Davis M, Calafat AM, Aldous KM. 2013. Population-based biomonitoring of exposure to organophosphate and pyrethroid pesticides in New York City. Environ Health Perspect 121:1349–1356; http://dx.doi.org/10.1289/ehp.1206015

## Introduction

In New York City (NYC), building density and disrepair increase the likelihood of pest infestations, which can lead to a reliance on indoor use of insecticidal chemicals. By the 1980s, organophosphates were the most common class of insecticides used in the United States, having largely replaced persistent organochlorine products [U.S. Environmental Protection Agency (EPA) 2004, 2011]. Organophosphates were used indoors in NYC until around 2000–2001, when structural pest control practice began shifting toward greater use of pyrethroid products [Horton et al. 2011; New York State (NYS) Department of Environmental Conservation (DEC) and Department of Health (DOH) 2011].

Both organophosphates and pyrethroids work by disrupting an insect’s nervous system. Organophosphates inhibit acetylcholinesterase, the enzyme that breaks down the neurotransmitter acetylcholine. Pyrethroids disrupt voltage-sensitive sodium channels in nerve cell membranes, similar to some older-generation organochlorine insecticides (Coats 1990; Shafer et al. 2005). Organophosphates are acutely toxic at high doses, and both occupational and nonoccupational exposures have been associated with adverse reproductive and neurodevelopmental outcomes in epidemiological studies (Hanke and Jurewicz 2004; Koureas et al. 2012). Although epidemiological studies of pyrethroids are fewer and less consistent than those of organophosphates, the combined human and animal literature suggests that pyrethroid exposures may also adversely affect the reproductive system and developing nervous system (Hanke and Jurewicz 2004; Koureas et al. 2012; Shafer et al. 2005).

Evidence that organophosphate exposure may be hazardous to a developing child led the U.S. EPA and manufacturers to reach agreement in 2000–2001 to phase out almost all indoor use of two common organophosphate insecticides, chlorpyrifos and diazinon (U.S. EPA 2000, 2001). These two insecticides, along with malathion (which is not registered for indoor residential use), had previously been the most commonly used insecticidal chemicals nationwide (U.S. EPA 2004, 2006). The reported use of organophosphates decreased from about 15% of total commercial insecticide applications in NYC by weight in 1999 to 3% (~ 7,000 pounds) in 2004, with almost all products being outdoor formulations [NYC Department of Health and Mental Hygiene (DOHMH) 2012; NYS DEC and DOH 2011]. Declines in levels of chlorpyrifos biomarkers were also reported in longitudinal data from a cohort of pregnant women in NYC followed from 2001–2004 (Whyatt et al. 2009).

Organophosphates are still commonly used in agriculture, and they accounted for approximately 40% (46 million pounds) of all insecticide products used nationwide in 2004 (U.S. EPA 2011). Most organophosphates in use today are either dimethyl (e.g., malathion and dimethoate) or diethyl (e.g., chlorpyrifos and diazinon) derivatives, which are enzymatically transformed or spontaneously hydrolyzed to dialkylphosphate (DAP) metabolites (Barr et al. 2004). Products containing pyrethroids are more frequently used in and around dwellings and on pets, and some active ingredients are used in agriculture (Spurlock and Lee 2008). Many pyrethroids are enzymatically transformed into the relatively non–class-specific metabolite 3-phenoxybenzoic acid (3-PBA), and some are also simultaneously transformed into one of several other metabolites, including *cis* or *trans*-3-(2,2-dichlorovinyl)-2,2-dimethylcyclopropane carboxylic acid (*cis-* or *trans*-DCCA) (e.g., permethrin, cypermethrin) or *cis*-3-(2,2-dibromovinyl)-2,2-dimethylcyclopropane carboxylic acid (*cis*-DBCA) (e.g., deltamethrin). Cyfluthrin is a relatively common pyrethroid that is transformed to 4-fluoro-3-phenoxybenzoic acid (4-F-3PBA) rather than 3-PBA, and simultaneously transformed to *cis-* or *trans*-DCCA (Barr 2010).Pyrethroid and organophosphate metabolites are excreted in urine.

Pesticide exposure occurs from dietary and nondietary ingestion, dermal absorption, and inhalation. Residential broadcast spraying is an important source of nondietary exposure in NYC, where confined indoor space and limited outdoor space increase the potential for contact with residual chemicals (Landrigan et al. 1999). Risk of exposure is amplified indoors because compounds are slower to degrade when they are not subject to sunlight, rain, and soil microbial activity (Simcox et al. 1995). Diazinon and chlorpyrifos were detected in all indoor air samples collected from a sample of NYC homes (Whyatt et al. 2007) and from the majority of floor-wipe samples in a national study of U.S. homes (Stout et al. 2009) several years after these compounds were phased out for indoor use.

In 2004, we conducted the first NYC Health and Nutrition Examination Survey (NYC HANES) modeled after the National Health and Nutrition Examination Survey (NHANES), which is an ongoing, population-based, cross-sectional survey of the health and nutrition status of residents of the United States [Centers for Disease Control and Prevention (CDC) 2013a]. The NYC HANES included a biomonitoring component to evaluate pesticide exposures by measuring urinary concentrations of organophosphate and pyrethroid metabolites. Local biomonitoring was conducted under the premise that the relative importance of population exposures and their sources may vary according to cultural, behavioral, and built environment characteristics of an area. Pesticide biomonitoring was also one component of comprehensive pesticide use, exposure, and outcome surveillance efforts by the NYC Health Department (Kass et al. 2004). Using results from the NYC HANES, we describe the distribution of urinary organophosphate and pyrethroid pesticide metabolite concentrations and their predictors in NYC adults in 2004.

## Methods

*Sample selection*. The 2004 NYC HANES was a population-based, cross-sectional survey representing the civilian, noninstitutionalized adult population (≥ 20 years of age) residing in the five boroughs (counties) of NYC (Manhattan, the Bronx, Brooklyn, Queens, and Staten Island). It was conducted between June and December 2004. Participants were recruited into the study using a three-stage cluster-sampling design: *a*) selection of census blocks, or groups of blocks; *b*) enumeration and random selection of households within selected areas; and *c*) random selection of household occupants. The target sample size was 2,000.

*Data collection*. Selected individuals were invited to any of four clinics in the boroughs of Manhattan, Brooklyn, the Bronx, or Queens for a study interview and examination (including urine collection and measurement of body weight). Using a face-to-face, computer-assisted personal interview, study participants were asked their age, sex, race/ethnicity [white, black/African American, Asian/Hawaiian/Pacific Islander (henceforth referred to as “Asian”), native American/Alaskan native, or other (henceforth referred to as “other”), and whether they consider themselves to be Hispanic/Latino], annual family income, education, and place of birth. Participants were asked about personal and professional pesticide use in the home in the past 3 months. They were also asked how often per day, week, month, or year they ate fruits and vegetables during the past 12 months, separated into questions about total fruit, “dark green” vegetables (henceforth referred to as “green vegetables”), and “other” vegetables (henceforth referred to as “non-green vegetables”). Separately, they were asked how often they ate any fruits or vegetables labeled “organic”’ or “chemical- or pesticide-free.” The survey instrument was translated into Spanish; interviews in other languages were conducted by translating the English language survey instrument with help from a staff member or family member who spoke both languages, or by using a telephone service that translated the English language questions as they were read over the phone (LanguageLine Solutions; http://www.languageline.com). Of the 3,634 eligible survey participants who were selected, 1,999 completed the interview and at least one component of the examination, which yielded an overall survey response of 55%.

Spot urine specimens (in 250-mL plastic containers with a lid) were provided by 1,832 participants (92%), yielding an overall response rate of 51% for this analysis. Specimens were immediately aliquotted and shipped on dry ice to the CDC, National Center for Environmental Health (NCEH) and the Wadsworth Laboratory at the NYS DOH, where they were frozen at –70°C until a random sample of specimens were analyzed in 2009–2010. NHANES samples have been stored similarly, and quality control (QC) data demonstrate stability over time (Barr DB, personal communication). Both laboratories are certified under the federal Clinical Laboratory Improvements Amendments of 1988 (CLIA 1988). A pilot analysis of organophosphate and pyrethroid metabolites conducted on a separate simple random sample of 380 NYC HANES specimens by the CDC in 2005 demonstrated feasibility of the present study (Kass 2009).

The NYC HANES protocol was approved by the NYC DOHMH and the NYS DOH institutional review boards. The involvement of the CDC laboratory was determined not to constitute engagement in human subject research. Study participants provided written, informed consent, and those who provided interview and laboratory data were remunerated $100. Additional information on data collection and protocols, as well as a detailed description of the study design, has been published elsewhere (Thorpe et al. 2006).

*Laboratory methods*. In 2009, the Wadsworth laboratory measured concentrations of six DAP metabolites [dimethylphosphate (DMP), diethylphosphate (DEP), dimethylthiophosphate (DMTP), diethylthiophosphate (DETP), dimethyldithiophosphate (DMDTP), and diethyldithiophosphate (DEDTP)] in a random sample of 883 of 1,832 available urine specimens, using high-performance liquid chromatography-isotope dilution tandem mass spectrometry (HPLC/MS/MS), with atmospheric pressure chemical ionization in negative-ion detection mode (Draper et al. 2008). Aliquots of urine (100 μL) were diluted, spiked with labeled internal standards, and directly injected onto a weak anion-exchange HPLC column to separate the six metabolites eluted with a water/methanol gradient containing 50 mM ammonium formate. Each analytical run included 20 NYC HANES samples, 3 QC materials (low, medium, and high concentration), 9 urine-based calibration standards, and 1 matrix blank. The QC concentrations in each run were evaluated using standard statistical probability rules (Westgard et al. 1981). The percent relative standard deviation of the QC results ranged from 6.3 to 20.3, depending on the concentration and analyte. The limits of detection (LODs) were 0.5 μg/L (DMP), 0.4 μg/L (DMTP), 0.2 μg/L (DEP), and 0.1 μg/L (DETP, DMDTP, and DEDTP). The total organophosphate metabolite concentration (ΣDAP), total diethyl alkylphosphate (ΣDEAP), and total dimethyl alkylphosphate (ΣDMAP) were calculated by first dividing each metabolite concentration by its molecular weight (154, 170, 186, 126, 132, and 158 for DEP, DETP, DEDTP, DMP, DMTP, and DMDTP, respectively), summing transformed metabolite concentrations, and multiplying the sum by 1,000 to obtain units of nanomoles per liter.

In 2009–2010, the NCEH laboratory measured concentrations of five metabolites of pyrethroid insecticides (3-PBA, 4-F-3-PBA, *cis*-DBCA, *trans*-DCCA, and *cis*-DCCA) in a random sample of 1,452 of the available urine specimens. The metabolites were first extracted from 1 mL of urine using semiautomated 96-well plate technology and 30-mg cartridges of a mixed-mode solid-phase extraction sorbent. The final extract (40 μL) and injection (10 μL) volumes were adjusted to achieve a 4.0-fold total concentration factor before analysis by using HPLC/MS/MS. Each analytical run included 36 NYC HANES samples; 3 QC materials of low, medium, and high concentration; 12 urine-based calibration standards; and 1 matrix blank. The QC concentrations in each run were evaluated using standard statistical probability rules (Caudill et al. 2008). The *cis*-DCCA results did not meet all quality assurance/QC guidelines and therefore are not reported. For most analytes in this study, the percent relative standard deviation of the QC results ranged from 6.2 to 17.6, depending on the concentration and the analyte. However, for *cis*-DBCA, the range was greater (23.6–29.9) because we lacked an isotope-labeled internal standard for this analyte. The LODs were 0.35 μg/L (4-F-3-PBA), 0.22 μg/L (*cis*-DBCA), 1.39 μg/L (*trans*-DCCA), and 0.64 μg/L (3-PBA). In 2010, the NCEH laboratory successfully participated in the German External Quality Assessment Scheme (Institute and Out-Patient Clinic for Occupational, Social and Environmental Medicine of the University Erlangen-Nuremberg 2009) for all the reported pyrethroid metabolites.

To account for variable dilution in urine, the NCEH laboratory measured creatinine in urine specimens selected for analysis of pyrethroid metabolites using an enzymatic reaction on a Roche Hitachi 912 chemistry analyzer (Roche Hitachi, Basel, Switzerland). The Wadsworth Laboratories measured creatinine in specimens selected for analysis of organophosphate metabolites using an enzymatic reaction on a Roche/Hitachi cobas c 501 analyzer. Pesticide metabolite concentrations are presented both as uncorrected (micrograms per liter) and corrected for creatinine (micrograms per gram creatinine).

*Statistical methods*. We calculated 50th and 95th percentiles of urinary metabolite concentrations and their 95% confidence intervals (CI) using a published method (Korn and Graubard 1998). To adjust for differential selection probabilities, survey nonresponse, and random selection of a subset of specimens for this analysis, we applied sample weights to all estimates. Weighting ensures that estimates reflect the age, sex, race/ethnicity, and borough of residence composition of the NYC population (U.S. Census Bureau 2004). Spearman correlation coefficients (*r*_S_) (unweighted) were calculated for combinations of related metabolites, with analyses limited to pairs of samples for which values were detectable for each sample. We conducted all statistical analyses using SAS version 9.3 (SAS Institute Inc., Cary, NC) and SUDAAN version 11 (Research Triangle Institute, Research Triangle Park, NC) to account for the complex sampling design.

To compare pesticide metabolite concentrations across levels of predictors, we calculated least-squares geometric means (LSGM) from multivariable linear regression models that included all predictors specified *a priori* and categorized as follows: age (20–39, 40–59, or ≥ 60 years), race/ethnicity (non-Hispanic black, white, or Asian; or Hispanic), sex (male or female), place of birth (U.S.-born or non–U.S.-born), annual income (< $20,000, $20,000 to < $50,000, $50,000 to < $75,000, or ≥ $75,000), education (less than high school degree, high school degree, some college, or college degree or higher), body weight (< 59 kg, 59 kg to < 68 kg, 68 kg to < 77 kg, 77 to < 88 kg, or ≥ 88 kg), personal pesticide use (yes or no), and professional pesticide use (yes or no). Dietary predictors were parameterized into categories that were defined so that participants were distributed approximately equally while retaining meaningful increments in consumption frequency: Fruit consumption, green vegetable consumption, and non-green vegetable consumption were each categorized as 0–2, 3–6, 7, or ≥ 8 times/week; organic fruit or vegetable consumption was categorized as 0, > 0 but < 2, or ≥ 2 times/week. We removed predictors from the final model if they had an associated beta coefficient *p*-value > 0.20 in a model that assigned ordinal scores to variables that were ordinal in nature (age, income, education, body weight, and fruit and vegetable consumption). We kept creatinine in all models as a predictor to adjust for variable dilution in spot urine samples, paramterized as the natural log of continuous creatinine values, because this transformation produced the best model fit. We forced age, race/ethnicity, and sex in all models because we wanted to present adjusted LSGMs across categories of these variables. Urinary metabolite concentrations below the LODs were assigned a value equal to the LOD divided by the square root of two. The *p*-values for predictors from final multivariable models are derived from a model that assigned ordinal scores to categories of the previously specified variables that were ordinal in nature.

We used ΣDAP to model organophosphate exposures and 3-PBA to model pyrethroid exposures. The pyrethroid metabolites should not be summed because some parent compounds may be metabolized into two of the measured metabolites. Thus, summing would result in an overestimation of exposure. Furthermore, 3-PBA is considered a good overall nonspecific indicator of pyrethroid exposures because it is a metabolite of several of the most commonly used pyrethroid parent compounds (Barr et al. 2010). In contrast, organophosphate pesticides are metabolized into just one of the measured metabolites. Measuring ΣDAP is a common approach to quantifying total exposure to organophosphates (Barr et al. 2004). We compared model results for ΣDAP against results for ΣDMAP and patterns were similar, so we have opted to present the more inclusive measure.

## Results

Among organophosphate metabolites, DEP was most likely to be detected (detected in 57% of urine samples) (Table 1). However, dimethyl metabolites were measured in higher concentrations than the diethyl compounds at the 95th percentiles of the distributions, with DMP and DMTP present at the highest concentrations (87.4 μg/L; 95% CI: 67, 103 μg/L and 74.7 μg/L; 95% CI: 41.9, 121 μg/L, respectively). In the subset of study participants 20–59 years of age (calculated for later comparison to national estimates), estimates were slightly lower (82.7 μg/L; 95% CI: 63, 102 μg/L and 55.6 μg/L; 95% CI: 38.7, 86.9 μg/L, respectively). Median concentrations of all metabolites except DEP were below their respective LODs. DMTP and DMDTP were the most strongly correlated organophosphate metabolites (*r*_S_ = 0.70), based on detectable measurements in 208 specimens. Other pairwise *r*_S_ values were < 0.35 (data not shown). The dimethyl metabolites contributed more than half of the ΣDAP concentration for > 80% of participants.

**Table 1:** Population-weighted percentiles of uncorrected and creatinine-corrected urinary concentrations of dialkyl phosphates in the NYC HANES 2004 study population.

Metabolite	*n*	Percent detects^*a*^	50th percentile (95% CI)		95th percentile (95% CI)	
			Uncorrected (μg/L)	Creatinine corrected (μg/g)	Uncorrected (μg/L)	Creatinine corrected (μg/g)
DMP (dimethylphosphate)	881	47.1	< LOD	< LOD	87.40 (67.00, 103.00)	88.65 (66.16, 105.00)
DMTP (dimethylthiophosphate)	881	44.4	< LOD	< LOD	74.70 (41.90, 121.00)	45.78 (28.20, 73.24)
DMDTP (dimethyldithiophosphate)	883	27.9	< LOD	< LOD	17.10 (8.13, 27.30)	8.73 (7.07, 18.21)
DEP (diethylphosphate)	883	57.0	0.79 (0.28, 1.31)	0.52 (0.35, 0.78)	11.90 (10.80, 14.30)	11.78 (8.55, 14.31)
DETP (diethylthiophosphate)	880	18.5	< LOD	< LOD	3.28 (2.17, 4.08)	1.89 (1.31, 2.69)
DEDTP (diethyldithiophosphate)	883	5.6	< LOD	< LOD	0.10 (< LOD, 1.53)	0.54 (0.31, 1.20)
^***a***^LODs were 0.5, 0.4, 0.1, 0.2, 0.1, and 0.1 μg/L for DMP, DMTP, DMDTP, DEP, DETP, and DEDTP, respectively.						

As noted elsewhere (Barr et al. 2005), creatinine concentration was associated with individual characteristics such as age, race/ethnicity, and sex (see Supplemental Material, Table S1). This resulted in a tendency for creatinine correction by division to modify patterns in ΣDAP concentrations across categories of these predictors (Table 2). For example, because males tend to have higher creatinine concentrations than females, correcting for creatinine by division generated a lower estimate of ΣDAP concentration in males relative to females, in contrast with the higher estimate in males generated by uncorrected data (Table 2). However, both population-weighted crude and creatinine-corrected estimates of urinary ΣDAP and ΣDMAP concentrations at the 50th and 95th percentiles were higher in older age groups, consistent with estimated geometric mean (GM) concentrations of ΣDAP from the multivariable regression model that included creatinine and other covariates as predictors (89 nmol/L; 95% CI: 75, 107 nmol/L in those 20–39 years of age and 136 nmol/L; 95% CI: 97, 192 nmol/L in those ≥ 60 years of age) (Table 3). Patterns of ΣDAP were less consistent across categories of sex and race/ethnicity when comparing the population-weighted crude, creatinine-corrected, and multivariable regression results. We focused on adjusted LSGM estimates when comparing concentrations among subgroups (Barr et al. 2005), suggesting higher ΣDAP concentrations in males (109 nmol/L; 95% CI: 90, 132 nmol/L) than in females (95 nmol/L; 95% CI: 80, 113 nmol/L), and in non-Hispanic blacks (111 nmol/L; 95% CI: 85, 144 nmol/L) and whites (116 nmol/L; 95% CI: 90, 149 nmol/L) than in Hispanics (84 nmol/L; 95% CI: 69, 103 nmol/L). We estimated the 95th percentile of ΣDMAP in the subset of participants 20–59 years of age (968 nmo/L; 95% CI: 795, 1,447 nmol/L) for later comparison with NHANES estimates.

**Table 2:** Population-weighted percentiles of uncorrected and creatinine-corrected urinary ∑DAP, ∑DMAP, and ∑DEAP concentrations in the NYC HANES 2004 study population.

Analytes/characteristic	*n*	50th percentile (95% CI)		95th percentile (95% CI)	
		Uncorrected (nmol/L)	Creatinine corrected (nmol/g)	Uncorrected (nmol/L)	Creatinine corrected (nmol/g)
∑DAP
All	876	114.92 (93.77, 141.53)	83.09 (66.53, 101.36)	1321.8 (870.18, 1739.1)	1003.9 (867.34, 1411.3)
Sex
Male	345	122.04 (91.81, 184.47)	81.21 (48.78, 123.43)	1496.4 (897.03, 1665.3)	1003.7 (857.54, 1532.5)
Female	531	108.14 (86.29, 130.50)	85.04 (68.03, 101.52)	1180.2 (743.40, 2245.1)	1049.0 (774.35, 1622.9)
Age (years)
20–39	434	91.13 (70.31, 118.45)	57.43 (44.45, 81.21)	1044.0 (720.09, 1601.7)	918.67 (585.87, 1069.2)
40–59	331	108.94 (81.96, 144.93)	91.67 (60.19, 123.43)	1321.8 (808.58, 1665.3)	1265.6 (826.06, 1839.9)
≥ 60	111	192.50 (115.42, 342.43)	170.42 (79.25, 266.95)	1496.4 (640.05, 3534.5)	910.18 (703.45, 1781.4)
Race/ethnicity^*a*^
White, non-Hispanic	239	135.26 (93.54, 190.02)	121.85 (76.72, 171.45)	1942.3 (865.06, 3534.5)	1532.5 (1003.9, 2207.2)
Black, non-Hispanic	186	114.92 (82.86, 174.68)	77.86 (44.91, 98.73)	967.28 (777.27, 1407.7)	897.37 (472.21, 1370.1)
Asian, non-Hispanic	116	69.79 (42.37, 123.12)	61.93 (31.60, 137.04)	626.53 (350.94, 868.10)	995.91 (398.00, 2657.4)
Hispanic	318	102.77 (73.99, 140.96)	74.65 (50.41, 91.70)	803.61 (674.82, 1439.0)	850.59 (467.63, 1024.8)
∑DMAP
All	879	83.54 (65.70, 110.44)	57.82 (40.77, 75.34)	1134.8 (825.06, 1542.3)	992.59 (855.55, 1334.2)
Sex
Male	347	110.23 (69.65, 140.69)	61.54 (38.89, 103.39)	1346.7 (859.01, 1542.3)	992.59 (836.71, 1516.1)
Female	532	76.40 (52.42, 95.79)	55.07 (39.73, 72.98)	1082.0 (687.94, 2113.8)	982.60 (700.78, 1554.3)
Age (years)
20–39	435	62.11 (51.56, 78.59)	38.52 (26.67, 55.35)	898.65 (657.89, 1542.3)	883.54 (512.43, 1040.6)
40–59	333	86.54 (57.76, 128.78)	62.05 (38.22, 90.25)	1225.1 (789.89, 1542.3)	1169.3 (707.00, 1746.7)
≥ 60	111	172.96 (92.05, 315.28)	153.25 (61.03, 235.41)	1494.7 (605.47, 3478.0)	870.89 (700.78, 1779.4)
Race/ethnicity^*a*^
White, non-Hispanic	240	101.95 (62.11, 159.98)	94.33 (42.04, 145.83)	1855.3 (868.01, 3478.0)	1287.6 (921.01, 2194.6)
Black, non-Hispanic	187	86.91 (54.69, 140.69)	52.64 (28.39, 83.67)	880.07 (712.11, 1494.7)	855.55 (503.10, 1389.3)
Asian, non-Hispanic	117	53.94 (21.74, 98.62)	33.12 (21.00, 92.84)	603.55 (349.22, 843.86)	885.40 (392.12, 2614.3)
Hispanic	318	80.04 (58.15, 110.23)	48.82 (34.89, 71.60)	771.64 (635.92, 1320.1)	828.69 (403.53, 1024.1)
∑DEAP
All	880	9.61 (7.77, 12.53)	6.41 (5.03, 8.18)	97.19 (83.26, 108.83)	90.00 (73.57, 120.90)
Sex
Male	347	5.10 (1.71, 9.56)	3.88 (2.84, 5.33)	84.62 (69.97, 113.09)	58.17 (36.64, 141.66)
Female	533	13.13 (9.69, 17.03)	10.21 (7.50, 12.10)	102.74 (84.71, 117.72)	102.83 (83.94, 130.86)
Age (years)
20–39	435	11.77 (8.62, 14.69)	6.51 (4.95, 8.34)	93.82 (74.48, 119.38)	76.29 (54.27, 112.83)
40–59	333	9.43 (5.10, 14.63)	7.35 (5.03, 10.53)	102.74 (76.32, 119.63)	116.43 (65.57, 165.52)
≥ 60	112	7.77 (1.71, 13.13)	3.94 (2.91, 9.64)	96.47 (43.39, 126.77)	92.34 (39.27, 166.36)
Race/ethnicity^*a*^
White, non-Hispanic	242	8.95 (5.15, 13.13)	6.73 (4.26, 10.48)	104.04 (74.13, 154.69)	130.02 (65.26, 179.91)
Black, non-Hispanic	186	15.65 (9.61, 21.90)	8.86 (4.85, 12.95)	96.47 (74.82, 119.38)	55.97 (42.76, 71.33)
Asian, non-Hispanic	116	3.87 (1.71, 10.47)	4.19 (2.39, 7.41)	74.82 (50.64, 131.72)	120.90 (58.85, 199.14)
Hispanic	319	9.04 (4.61, 11.84)	5.33 (3.39, 6.84)	82.34 (65.59, 106.71)	84.69 (50.89, 112.83)
^***a***^Race/ethnicity categories exclude 17 participants who categorized themselves as “other.”					

**Table 3:** Adjusted LSGMs for urinary ∑DAP concentrations across study population characteristics, NYC HANES 2004.

Characteristic^*a*^	*n*^*b*^	Crude GM (nmol/L) (95% CI)	LSGM (nmol/L) (95% CI)^*c*^	*p*-Value^*d*^
Sex
Male	329	113 (92, 138)	109 (90, 132)
Female	519	92 (76, 112)	95 (80, 113)	0.24
Age (years)
20–39	417	86 (72, 103)	89 (75, 107)
40–59	325	98 (79, 122)	98 (79, 121)
≥ 60	106	147 (99, 217)	136 (97, 192)	0.03
Race/ethnicity
White, non-Hispanic	238	119 (91, 157)	116 (90, 149)	0.03
Black, non-Hispanic	183	110 (84, 144)	111 (85, 144)	0.18
Asian, non-Hispanic	114	73 (55, 97)	80 (60, 108)	0.69
Hispanic	313	85 (69, 104)	84 (69, 103)	Reference
Professional pest control
Yes	238	121 (91, 161)	124 (96, 160)
No	610	94 (80, 111)	94 (81, 109)	0.08
Weekly fruit servings
0–2	238	79 (63, 99)	74 (59, 94)
3–6	202	93 (69, 126)	91 (68, 122)
7	283	116 (91, 147)	118 (94, 149)
> 7	125	127 (89, 183)	141 (102, 195)	< 0.01
Weekly servings of non-green vegetables
0–2	205	96 (76, 122)	107 (84, 136)
3–6	285	119 (97, 145)	126 (103, 153)
7	266	101 (80, 128)	97 (78, 122)
> 7	92	70 (40, 123)	55 (32, 92)	0.02
^***a***^Age, sex, and race/ethnicity were forced into the model with other predictors specified *a priori* (see “Methods”) that were retained if the associated *p*-value was < 0.20. ^***b***^Participants with missing covariate values or of “other” race/ethnicity were excluded from multi­variable analyses. ^***c***^Model includes all predictors in the table simultaneously plus the natural log of creati­nine concentration. ^***d***^*p*-Values are associated with beta coefficients in a separate model that parameterizes age and fruit and non-green vegetable consumption as ordinal variables scored 1, 2, 3, and 4.				

Fruit consumption and professional pesticide use were both positively associated with urinary ΣDAP concentration in LSGM models (Table 3). The estimated adjusted LSGM urinary ΣDAP among those who ate fruit more than once per day (141 nmol/L; 95% CI: 102, 195 nmol/L) was higher than the corresponding estimate for those who ate no more than two servings per week (74 nmol/L; 95% CI: 59, 94 nmol/L), with evidence of a linear trend across categories (*p* < 0.01). The estimated adjusted LSGM urinary ΣDAP concentration was higher among those who reported professional pesticide use in the last 3 months (124 nmol/L; 95% CI: 96, 160 nmol/L) compared with other participants (94 nmol/L; 95% CI: 81, 109 nmol/L). Although those who consumed non-green vegetables > 7 times/week had lower estimated adjusted LSGM ΣDAP concentration (55 nmol/L; 95% CI: 32, 92 nmol/L) than those who consumed ≤ 2 servings/week (107 nmol/L; 95% CI: 84, 136 nmol/L), estimates did not decrease monotonically.

Among pyrethroid metabolites, we were most likely to detect the nonspecific metabolite 3-PBA (detected in 58.5% of urine samples) (Table 4). However, the estimated 95th percentile of urinary concentration of *trans*-DCCA was slightly higher (5.94 μg/L; 95% CI: 4.44, 8.17 μg/L) than that of 3-PBA (5.23 μg/L; 95% CI: 4.29, 6.26 μg/L). In the subset of study participants 20–59 years of age (calculated for later comparison to national estimates), estimates were slightly higher (6.11 μg/L; 95% CI: 4.62, 8.03 μg/L and 5.66 μg/L; 95% CI: 4.75, 6.53 μg/L, respectively). Differences in the estimated 95th percentiles of 3-PBA concentrations across categories of age, sex, and race/ethnicity were relatively small (Table 5). Similar to results for DAP metabolites, correcting for creatinine by division tended to lower estimated concentrations for population subgroups with higher average creatinine (males, younger age, non-Hispanic blacks, and Hispanics) and increase estimates for subgroups with lower average creatinine levels (females, older age, non-Hispanic whites, and Asians). The magnitude of differences in estimated LSGM concentrations across categories of predictors from a multivariable regression model was ≤ 0.31 μg/L, which is less than half of the 3-PBA LOD (Table 6).

**Table 4:** Population-weighted percentiles of uncorrected and creatinine-corrected urinary concentrations of pyrethroid metabolites in the NYC HANES 2004 study population.

Metabolite	*n*	Percent detects^*a*^	50th percentile (95% CI)		95th percentile (95% CI)	
			Uncorrected (μg/L)	Creatinine corrected (μg/g)^*b*^	Uncorrected (μg/L)	Creatinine corrected (μg/g)^*b*^
4-F-3PBA (4-fluoro-3-phenoxybenzoic acid)	1,452	8.1	< LOD	< LOD	0.44 (0.37, 0.52)	1.13 (1.00, 1.34)
*cis*-DBCA [*cis*-3-(2-2-dibromovinyl)-2-2-dimethyl propane carboxylic acid]	1,311	1.5	< LOD	< LOD	< LOD	< LOD
*trans*-DCCA [*trans*-3-(2-2-dichlorovinyl)-2-2-dimethyl propane carboxylic acid]	1,452	14.0	< LOD	< LOD	5.94 (4.44, 8.17)	6.66 (5.51, 8.29)
3-PBA (3-phenoxybenzoic acid)	1,452	58.5	0.76 (0.72, 0.81)	0.75 (0.69, 0.82)	5.23 (4.29, 6.26)	4.51 (3.89, 5.80)
^***a***^LODs correspond to 0.35, 0.22, 1.39, and 0.64 μg/L for 4-F-3PBA, *cis*-DBCA, *trans*-DCCA, and 3-PBA, respectively. ^***b***^Creatinine-corrected values are missing for two individuals.						

**Table 5:** Population-weighted percentiles of uncorrected and creatinine-corrected urinary 3‑PBA concentrations in the NYC HANES 2004 study population.

Characteristic	*n*	50th percentile (95% CI)		95th percentile (95% CI)	
		Uncorrected (μg/L)	Creatinine corrected (μg/g)^*a*^	Uncorrected (μg/L)	Creatinine corrected (μg/g)^*a*^
All	1,452	0.76 (0.72, 0.81)	0.75 (0.69, 0.82)	5.23 (4.29, 6.26)	4.51 (3.89, 5.80)
Sex
Male	602	0.76 (0.70, 0.82)	0.61 (0.55, 0.68)	4.70 (3.66, 6.34)	3.30 (2.94, 4.00)
Female	850	0.76 (0.71, 0.82)	0.89 (0.82, 0.99)	5.65 (4.33, 7.83)	6.00 (4.24, 8.81)
Age (years)
20–39	708	0.78 (0.72, 0.84)	0.69 (0.62, 0.79)	5.42 (4.05, 8.22)	4.08 (3.27, 5.22)
40–59	554	0.75 (0.69, 0.81)	0.83 (0.71, 0.89)	5.31 (3.71, 6.79)	4.59 (3.91, 7.42)
≥ 60	190	0.78 (0.45, 0.92)	0.76 (0.63, 1.00)	4.36 (2.56, 7.73)	4.97 (3.15, 16.58)
Race/ethnicity^*b*^
White, non-Hispanic	411	0.76 (0.65, 0.85)	0.83 (0.76, 0.94)	5.26 (3.66, 10.23)	6.00 (4.23, 13.78)
Black, non-Hispanic	315	0.87 (0.79, 0.95)	0.63 (0.55, 0.69)	5.50 (3.22, 9.41)	3.18 (2.66, 4.19)
Asian, non-Hispanic	180	0.88 (0.71, 1.02)	1.12 (0.98, 1.47)	6.26 (4.41, 10.89)	7.89 (4.45, 15.57)
Hispanic	519	0.67 (0.45, 0.73)	0.63 (0.56, 0.71)	4.05 (3.08, 5.42)	3.46 (2.57, 5.11)
^***a***^Creatinine-corrected values are missing for 2 participants. ^***b***^Race/ethnicity categories exclude 27 participants who categorized themselves as “other.”					

**Table 6:** Adjusted LSGMs for urinary 3‑PBA concentrations across study population characteristics, NYC HANES 2004.

Characteristic^*a*^	*n*^*b*^	Crude GM (μg/L) (95% CI)	LSGM (μg/L) (95% CI)^*c*^	*p*-Value^*d*^
Sex
Male	578	0.92 (0.86, 0.99)	0.87 (0.81, 0.93)
Female	826	0.93 (0.87, 1.01)	0.98 (0.91, 1.06)	0.02
Age (years)
20–39	683	0.95 (0.87, 1.02)	0.92 (0.85, 0.99)
40–59	538	0.92 (0.86, 0.99)	0.94 (0.88, 1.01)
≥ 60	183	0.90 (0.78, 1.04)	0.92 (0.80, 1.06)	0.88
Race/ethnicity
White, non-Hispanic	408	0.93 (0.84, 1.03)	0.95 (0.85, 1.05)	0.05
Black, non-Hispanic	308	0.99 (0.89, 1.11)	0.91 (0.82, 1.01)	0.21
Asian, non-Hispanic	176	1.08 (0.92, 1.26)	1.15 (0.99, 1.35)	< 0.01
Hispanic	512	0.82 (0.75, 0.90)	0.84 (0.76, 0.92)	Reference
Professional pest control
Yes	407	0.99 (0.89, 1.09)	0.98 (0.9, 1.08)
No	997	0.91 (0.85, 0.97)	0.91 (0.86, 0.96)	0.15
Weekly green vegetable servings
0–2	443	0.81 (0.75, 0.88)	0.83 (0.77, 0.90)
3–6	459	0.98 (0.89, 1.07)	0.97 (0.89, 1.05)
7	345	0.91 (0.83, 0.99)	0.91 (0.84, 1.00)
> 7	157	1.16 (0.94, 1.44)	1.12 (0.91, 1.36)	0.01
Education
< High school	417	0.88 (0.80, 0.96)	0.89 (0.81, 0.99)
High school graduate	260	0.91 (0.79, 1.05)	0.89 (0.77, 1.02)
Some college	303	0.85 (0.78, 0.93)	0.85 (0.78, 0.92)
≥ College degree	424	1.04 (0.94, 1.14)	1.03 (0.93, 1.15)	0.11
^***a***^Age, sex, and race/ethnicity were forced into all models; other predictors were specified *a priori* (see “Methods”) and retained if the associated *p*-value was < 0.20. ^***b***^Participants with missing covariate values or of “other” race/ethnicity were excluded from all multi­variable analyses. ^***c***^Model includes all predictors simultaneously plus the natural log of creati­nine concentration. ^***d***^*p*-Values are associated with beta coefficients in a separate model that parameterizes age, green vegetable consumption, and education as ordinal variables scored 1, 2, 3, or 4.				

3-PBA and *trans*-DCCA were highly correlated (*r*_S_ = 0.98, based on 189 samples with detectable values for both metabolites), whereas 3-PBA and 4-F-3PBA were not correlated (*r*_S_ = 0.09, based on 106 samples). We did not calculate correlations among other pairs of metabolites because the sample sizes (number of detectable values) were too small.

## Discussion

We measured metabolites of organophosphates and pyrethroids in urine from a representative sample of adult NYC HANES participants to better understand pesticide exposure patterns in NYC.

**Figure 1:**
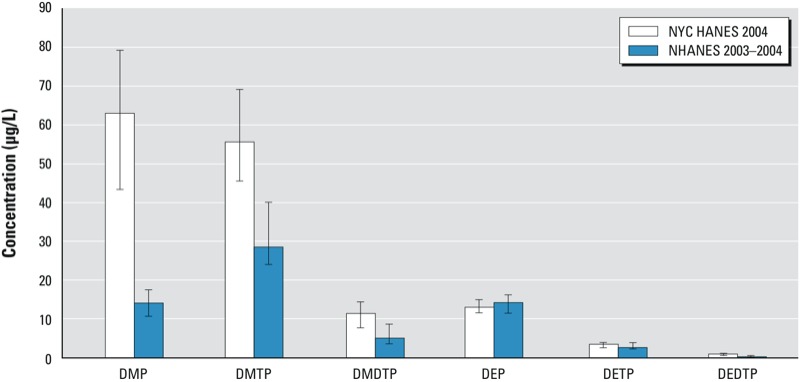
Comparison of 95th percentiles and 95% CIs for urinary organophosphate metabolite concentrations in adults 20–59 years of age in NYC (NYC HANES 2004; present study) and nationwide (NHANES 2003–2004; CDC 2013b).

Levels of urinary dimethyl organophosphate metabolites were higher in NYC than nationwide among those most exposed (at the 95th percentiles) (CDC 2013b) (Figure 1). The estimated 95th percentiles of urinary DMP and DMTP concentration in a subset of our data limited to adults 20–59 years of age (82.7 μg/L and 55.6 μg/L, respectively) were higher than national estimates in the same age-limited subset from NHANES 2003–2004 (14.1 μg/L and 28.5 μg/L, respectively) (CDC 2013b). (Note, however, that the national estimate for the DMP 95th percentile increased to 30.3 μg/L in NHANES 2007–2008.) Similarly, we estimated a higher 95th percentile for ΣDMAP in the same subset limited to 20- to 59-year-olds (968 nmol/L) compared with a report from NHANES 1999–2000 (426 nmol/L) (Barr et al. 2004). In Canada, the 95th percentile estimates for dimethyl metabolites from population-based biomonitoring conducted in 2007–2009 among children and adults combined were also lower than in the NYC HANES 2004 (25 μg/L and 87.4 μg/L, 40 μg/L and 74.7 μg/L, and 6 μg/L and 17.1 μg/L for DMP, DMTP, and DMDTP, respectively) (Haines and Murray 2012). In urban Frankfurt/Main, Germany, in 1998 (Heudorf and Angerer 2001a), 95th percentile estimates for adults ≥ 20 years of age were mostly higher (102.5 μg/L, 125.8 μg/L, and 13.1 μg/L for DMP, DMTP, and DMDTP, respectively) than in NYC.

The dimethyl metabolites are derived from pesticides that are most commonly used in agriculture, such as azinophos methyl, phosmet, and malathion; residues from this class of active ingredients were detected in 41% of apple, pear, and strawberry samples measured by the U.S. Department of Agriculture (USDA) Pesticide Data Program in 2004 (USDA 2006). Thus, our observation of higher ΣDAP concentrations associated with higher fruit consumption in the NYC HANES is not surprising. However, we found no published evidence that more frequent fruit consumption in NYC might explain higher exposure than in the nation as a whole.

Estimates of urinary concentrations of diethyl metabolites in NYC in a subset of our data limited to adults 20–59 years of age were similar to national NHANES 2003–2004 estimates for the same age-specific subset (Figure 1); this result was not surprising because the most widely used parent compounds—chlorpyrifos and diazinon—were phased out for residential use starting in 2000. Estimates for NYC were also similar, or slightly lower, than estimates from biomonitoring studies from Canada and Germany (Haines and Murray 2012; Heudorf and Angerer 2001a). Chlorpyrifos can persist in indoor environments (Stout et al. 2009; Whyatt et al. 2007), but exposures may have dissipated by 2004.

**Figure 2:**
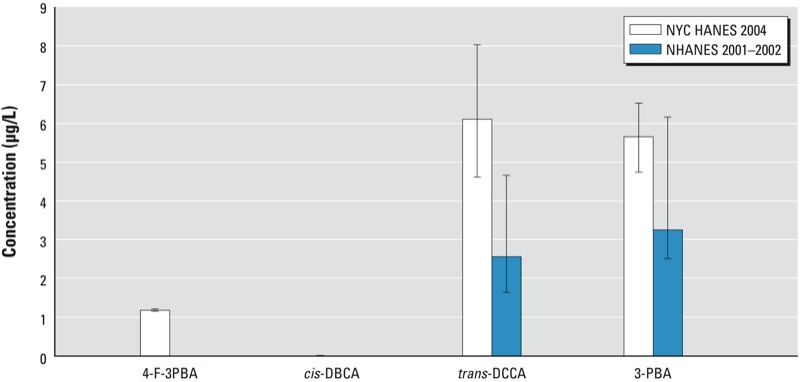
Comparison of 95th percentiles and 95% CIs for urinary pyrethroid metabo­lite concentrations in adults 20–59 years of age in NYC (NYC HANES 2004; present study) and nationwide [NHANES 2001–2002 (most recent year available for estimates of urinary pyrethroid metabolite concentrations); CDC 2009]. The absence of a bar indicates a 95th percentile < LOD.

Levels of urinary pyrethroid metabolites were higher in NYC than nationwide among those most exposed (at the 95th percentiles) (CDC 2009) (Figure 2). The estimated 95th percentiles of urinary *trans*-DCCA and 3-PBA concentrations in a subset of our data limited to adults 20–59 years of age (6.11 μg/L and 5.66 μg/L, respectively) were higher than the most recent national estimates (at the time of this writing) of *trans*-DCCA and 3-PBA in the subset of the same age from NHANES 2001–2002 (2.56 μg/L and 3.25 μg/L, respectively) (CDC 2009) (Figure 2). The 95th percentile estimate of urinary *trans*-DCCA among NYC adults of all ages (5.94 μg/L) was also higher than in urban Frankfurt, Germany, in 1998 (1.28 μg/L) (Heudorf and Angerer 2001b). (The German study did not measure 3-PBA.) Concentrations of 3-PBA were highly correlated with *trans*-DCCA (*r*_S_ = 0.98), suggesting that 3-PBA was more likely to be a marker of exposure to permethrin and cypermethrin (which metabolize to both 3-PBA and *trans*-DCCA) than other common pyrethroid products that metabolize only to 3-PBA (Barr et al. 2010). We estimated measurable levels of 4-F-3PBA (0.44 μg/L) at the 95th percentile of the exposure distribution in NYC, compared with an estimate below the detectable level in NHANES 2001–2002, even though NHANES had a lower LOD (0.2 μg/L) than did the present study (0.35 μg/L). This observation suggests greater exposure to cyfluthrin in NYC than in the United States as a whole. Cyfluthrin is registered for “professional use only” in New York State, but products containing this active ingredient have reportedly been used by the general public and found for sale illegally on NYC streets and in stores (NYC DOHMH 2012; Semple 2011).

The USDA Pesticide Data Program detected residues of pyrethroids in 43% of lettuce and spinach samples (but almost no fruit samples) in 2004 (USDA 2006). Thus, it is not surprising that more frequent consumers of green vegetables had higher concentrations of 3-PBA than infrequent consumers (Table 6). However, the differences in estimated LSGM 3-PBA concentrations across categories of green vegetable consumption were small.

In a study of children who were fed an organic diet, residential pesticide use was a stronger predictor of urinary pyrethroid metabolite concentrations than was dietary ingestion (Lu et al. 2006). In the present study, the difference in 3-PBA exposure among participants who reported professional pesticide use in the past 3 months was relatively small compared with that of those who did not, and personal pesticide use did not meet the criterion for inclusion in the final models. However, a single question on use in the past 3 months may have been a poor proxy for exposure because pyrethroid metabolites are eliminated in urine for only several days after exposure, and a single question might have been insufficient for capturing exposure in the more relevant period just prior to urine collection. People may also be exposed to pesticides unknowingly, after treatments throughout their buildings or elsewhere.

One of the rationales for conducting biomonitoring using a local HANES was the premise that the urban environment introduces exposure opportunities that differ from the nation as a whole. In NYC, characteristically small living areas that are densely situated in multiunit buildings allow single exposure sources to affect many people simultaneously. In a separate NYC HANES analysis of serum cotinine (a marker of exposure to environmental tobacco smoke) in nonsmokers, levels were higher than those estimated for the nation as a whole, even though the prevalence of smoking in NYC is lower (Ellis et al. 2009). This observation and ours are consistent with the hypothesis that urban housing characteristics can magnify exposure to some environmental chemicals. We did not collect data on housing characteristics or conditions, so we were unable to assess specific aspects of housing that may contribute to exposures. Our results are further limited by our reference to NYC as densely urban throughout, even though there are areas in the outer boroughs that have lower population and construction density.

Reported frequency of organic fruit or vegetable consumption did not meet the criterion for inclusion in our multivariable regression models of urinary ΣDAP or 3-PBA concentrations, and inclusion of this predictor in the models had little effect on the exposure patterns in Tables 3 and 6 (data not shown). However, it may have been difficult to derive an accurate measurement of organic produce consumption using data from the single question that was asked on this topic.

There may have been residual confounding of our estimates by behavioral factors that lead people to seek less contaminated produce or avoid exposure to pesticide applications. Unmeasured confounding may have contributed to or been responsible for the negative association between consumption of non-green vegetables and urinary ΣDAP concentrations. It is possible that those who consume vegetables frequently are also more likely to seek less-contaminated produce or avoid pesticide exposures, in general. Confounding of this sort may also explain the association between professional pest control and ΣDAP in our study population, because ΣDAP largely represents exposure to dimethyl pesticides, which are not typically used indoors. They are, however, readily available for sale to control fleas or ticks on pets, or for outdoor garden care. Those who report using professional pest control services may also be more likely to use organophosphate pesticide products on pets or outside their home.

We caution that positive associations between fruit and vegetable consumption and pesticide exposures reported here are based on differences in metabolite concentration that are relatively small and not likely to be biologically meaningful. The maximum difference in 3-PBA concentration across categories of predictors was 0.31 μg/L, which is about one-half of the LOD and may therefore represent random statistical variation. Furthermore, these associations need to be viewed in balance with the many nutritional benefits of fruits and vegetables. Another consideration is that we have measured concentrations of pesticide metabolites in urine, which are not necessarily indicative of exposure to the parent compounds. A person may be exposed to the pesticide itself or one of its degradation products (Lu et al. 2005).

For the purpose of comparing estimates across populations, we have most confidence in our estimates of 95th percentiles. Estimates of median concentrations for many of the metabolites we measured were below or near the LOD. We do not advise comparing estimates of the prevalence of detectable values across populations when different laboratories are used because detectable levels are a function of the analytic method rather than an objective measure of exposure. We also do not advise comparing creatinine-corrected estimates across populations because dividing by creatinine can produce false-negative associations with predictors of lean body mass, which may vary across populations or subgroups within a study (Barr et al. 2005).

Since 2004 when this study was conducted, the NYC health department has attempted to lessen the potential for unintended pesticide exposures by enacting local laws to prohibit city agency use of particularly hazardous chemicals and by promoting integrated pest management, which is less chemical-based, focusing instead on improving sanitary and structural conditions to deny pests food, water, harborage, and movement (City of New York 2005, 2013). The use of sprays and foggers spreads chemicals indiscriminately around the living area and potentially into neighboring spaces. At the high end of the distribution, our data suggest that exposure to pyrethroid and some organophosphate pesticides may be higher in NYC than in the United States overall, underscoring the importance of considering pest and pesticide burdens in cities when formulating pesticide use regulations.

## Supplemental Material

(147 KB) PDF
