# Supplementary material for: Population-Based Biomonitoring of Exposure to Organophosphate and Pyrethroid Pesticides in New York City
Source: Environ Health Perspect. 2013 Sep 27;121(11-12):1349–56. doi: 10.1289/ehp.1206015 (PMC3855501; doi:10.1289/ehp.1206015)
Supplement: (147 KB) PDF [file ehp.1206015.s001.508.pdf]

Supplemental Material

Population-Based Biomonitoring of Exposure to Organophosphate and Pyrethroid Pesticides in New York City

Wendy McKelvey, J. Bryan Jacobson, Daniel Kass, Dana Boyd Barr, Mark Davis, Antonia M. Calafat, and Kenneth M. Aldous

Supplemental Material, Table S1. Multiple linear regression of natural log of urinary creatinine concentration (µg/L) on participant characteristics using data from two analyses of urinary pesticide metabolites, NYC HANES, 2004

	Data from Organophosphate Metabolite Analysis (n=863)		Data from Pyrethroid Metabolite Analysis (n=1,420)	
Parameter	β (95% CI)	Pr > |t|	β (95% CI)	Pr > |t|
Intercept	4.657 (4.457, 4.858)	< 0.0001	4.532 (4.346, 4.718)	< 0.0001
Sex				
Female	-0.251 (-0.339, -0.163)	< 0.0001	-0.310 (-0.394, -0.217)	< 0.0001
Male (Reference)	0	--	0	--
Age (years)	-0.079 (-0.142, -0.017)	0.0127	-0.099 (-0.155, -0.043)	< 0.0001
Race/Ethnicity				
Hispanic	0.202 (0.109, 0.295)	< 0.0001	0.212 (0.110, 0.314)	< 0.0001
Asian, Non-Hispanic	0.003 (-0.142, 0.148)	0.9684	-0.014 (-0.157, 0.129)	0.8512
Black, Non-Hispanic	0.309 (0.196, 0.421)	< 0.0001	0.359 (0.244, 0.475)	< 0.0001
White, Non-Hispanic (Reference)	0	--	0	--
Body Weight (kg)	0.217 (0.157, 0.278)	< 0.0001	0.158 (0.101, 0.216)	< 0.0001
